# Effectiveness and Safety of Baricitinib for Juvenile Idiopathic Arthritis–Associated Uveitis or Chronic Anterior Antinuclear Antibody–Positive Uveitis

**DOI:** 10.1002/acr.25644

**Published:** 2026-02-01

**Authors:** Athimalaipet V. Ramanan, Catherine M. Guly, Gabriele Simonini, Stuart Y. Keller, Priyanka Sen, Thorsten Holzkaemper, Joana Araújo, Pierre Quartier

**Affiliations:** ^1^ Bristol Royal Hospital for Children and Translational Health Sciences Bristol United Kingdom; ^2^ Bristol Eye Hospital, University Hospitals Bristol and Weston NHS Foundation Trust Bristol United Kingdom; ^3^ ERN ReConnet Center, Meyer Children's Hospital IRCCS and University of Florence Florence Italy; ^4^ Eli Lilly and Company Indianapolis Indiana; ^5^ RAISE Reference Centre for Rare Diseases, Necker‐Enfants Malades Hospital, Assistance Publique‐Hopitaux de Paris and Université Paris‐cité Paris France

## Abstract

**Objective:**

To evaluate the efficacy and safety of baricitinib in pediatric patients with active juvenile idiopathic arthritis–associated uveitis (JIA‐U) or chronic anterior antinuclear antibody–positive uveitis, who had an inadequate response to methotrexate (MTX) or biologic disease‐modifying antirheumatic drugs (bDMARDs).

**Methods:**

JUVE‐BRIGHT was an open‐label, active‐controlled phase‐3 multicenter trial that used a novel design, including 1:1 randomization to an active reference arm. The primary efficacy endpoint was the proportion of responders at week 24 (W24), defined according to the Standardization of Uveitis Nomenclature (SUN) criteria as a two‐step decrease in the level of inflammation (anterior chamber cells) or decrease to zero through W24 in the most severely affected eye at baseline. Study success was based on a prespecified Bayesian success rule: the study was deemed successful if there was >80% posterior probability that the baricitinib SUN criteria response rate at W24 was at least 57%.

**Results:**

This study enrolled 30 pediatric patients. The study primary endpoint was not met. In the baricitinib group, 36.8% of MTX–inadequate responder (MTX‐IR) and bDMARD‐IR patients and 20% of MTX‐IR patients achieved a two‐step decrease in SUN criteria at W24. Eight patients (33.3%) achieved a response at W24, resulting in 1.03% posterior probability of a response rate of >57%. Safety data were consistent with the established safety profile in other baricitinib indications in pediatric and adult patients.

**Conclusion:**

Although the primary endpoint was not met, the data provide important information on baricitinib for the treatment of children with JIA‐U refractory to both MTX and bDMARDs. Baricitinib safety profile in this study was consistent with previous studies in children and adults with other diseases.

## INTRODUCTION

Juvenile idiopathic arthritis (JIA) is the most common rheumatic disease of childhood, with JIA‐associated uveitis (JIA‐U) being the most common extra‐articular manifestation of the disease. In patients with JIA, the prevalence of uveitis ranges from 11.6%^1^ to 30%.^2^ Uveitis is an inflammation of the uveal components of the eye, which includes the iris, choroid, and ciliary body.^3^ Despite the well‐documented link between JIA and uveitis, the reason for uveal inflammation is not well understood. It is likely that the pathophysiology of JIA‐U involves both genetic and environmental elements.^3^ A number of risk factors for JIA‐U have been identified, which include sex, JIA category, age at onset, and anterior antinuclear antibody (ANA) and HLA‐B27 positivity.^2^, ^4^, ^5^, ^6^ Children with chronic ANA‐positive uveitis without systemic features (rash, fever, generalized lymph node enlargement, hepatomegaly, splenomegaly, or serositis) have a similar clinical course and treatment response to children with a diagnosis of JIA‐U.^7^

**SIGNIFICANCE & INNOVATIONS**

- This study investigates baricitinib treatment for children with juvenile idiopathic arthritis–associated uveitis or chronic antinuclear antibody–positive uveitis who are inadequate responders (IRs) to methotrexate (MTX) or IRs to both MTX and biologic disease‐modifying antirheumatic drugs.
- The study design used is the first adaptive design trial with a comparator arm in a rare disease in children, which may be beneficial for future pediatric studies.
- This study presents the use of a novel Bayesian design to overcome recruitment challenges for a rare pediatric disease.

The first line of treatments for JIA‐U is topical glucocorticoids. Following failure of adequate control of inflammation of topical glucocorticoids or in children with severe uveitis or ocular complications, the use of disease‐modifying antirheumatic drugs (DMARDs) are employed. Methotrexate (MTX) is the first‐line systemic therapy.^6^ However, patients with moderate to severe JIA‐U can be refractory to MTX, and for these patients, biologic DMARDs (bDMARDs) are initiated.^8^, ^9^, ^10^ Adalimumab, an anti–tumor necrosis factor alpha (anti‐TNFα) monoclonal antibody, is the only licensed biologic therapy for JIA‐U. Although there is a high rate of primary treatment response with adalimumab in JIA‐U^11^, ^12^ and uveitis control with adalimumab is greater than infliximab and etanercept in the clinical setting,^13^ discontinuation of treatment has been reported due to reactivation of uveitis and development of anti‐adalimumab antibodies.^14^, ^15^ Furthermore data from the ORCHIDEA registry reported 40% of JIA‐U patients receiving adalimumab did not achieve clinical remission during a two‐year treatment period.^16^ Therefore, in patients that do not respond to MTX and anti‐TNF drugs, new therapeutic approaches are required. Recently, an anti–interleukin‐6 receptor, tocilizumab, has been investigated as a therapeutic with MTX in anti‐TNF refractory JIA‐U patients. Although the prespecified criterion was not met, 33% had a two‐step improvement in the level of inflammation (anterior chamber [AC] cells) at week 12, and a further 14% had a one‐step improvement at week 24.^17^ This highlights the lack of alternative bDMARDs to treat JIA‐U and the need to investigate different mechanisms of action.

Case reports and small series of patients have suggested a benefit of JAK inhibitors, including baricitinib, in adult patients with JIA‐U.^18^, ^19^, ^20^, ^21^ Baricitinib has demonstrated clinical safety and efficacy in adult patients with moderate to severe active rheumatoid arthritis (RA) in phase 3 studies,^22^, ^23^ as well as in pediatric patients with polyarticular course JIA.^24^ As the etiology and pathogenesis of JIA, JIA‐U, and ANA‐positive uveitis are still poorly understood but share several immunologic abnormalities identified in RA, it is hypothesized that baricitinib may inhibit JAK enzymatic activity associated with JIA‐U and ANA‐positive uveitis pathogenesis, thus providing a novel therapeutic approach.^7^ Therefore, the aim of this study is to evaluate the efficacy and safety of oral baricitinib administered once daily to pediatric patients with JIA‐U or chronic ANA‐positive uveitis without systemic features who had an inadequate response to topical steroids, as well as MTX or bDMARDs. Additionally, this study presents a novel approach to the study design of pediatric trials, including the addition of a reference arm and the use of Bayesian analysis.

## MATERIALS AND METHODS

### Study design

JUVE‐BRIGHT was an open‐label, active‐controlled phase 3 trial conducted at 23 centers, including children from 2 to <18 years old with JIA‐U or chronic ANA‐positive uveitis without systemic features (ie, rash, fever, generalized lymph node enlargement, hepatomegaly, splenomegaly, or serositis) who had an inadequate response to topical steroids, as well as MTX or bDMARDs. The study protocol has been previously published including study design and eligibility criteria.^7^ Briefly, patients were eligible to enroll in the study only if they met several criteria at screening and at baseline, including the following:

1. Were at least 2 years and less than 18 years of age; full date of birth was collected except in countries in which it is not allowed.
2. Had a diagnosis of JIA‐U or chronic ANA‐positive uveitis without systemic features.
3. Had active anterior uveitis, defined as cellular infiltrate in the AC of Standardization of Uveitis Nomenclature (SUN) criteria grade ≥1+ at visit 1 (screening) and visit 2 (potential randomization), despite prior treatment with adequate doses of topical steroid therapy and MTX.
4. Had an inadequate response or intolerance to MTX (minimum dose of 10 mg/m^2^/week, with a maximum dose of 25 mg/m^2^/week). Patients considered to have an inadequate response must have received MTX for at least 12 weeks before an inadequate response could have been determined and must have been on a stable dose for at least 4 weeks before screening if continuing MTX therapy during the study.
5. Had been receiving topical glucocorticoid eye drops at a stable dose for at least two weeks before screening (maximum of four drops/day per eye at screening).

Individuals who were taking or who might have been taking JIA or JIA‐U medications or treatments interfering with the ability to assess the safety and efficacy of baricitinib were excluded from the study.

At baseline, patient race was self‐reported from a fixed set of categories. This study includes adalimumab in the form of an active‐control reference arm. The aim was to recruit at least 20 patients who had an inadequate response or intolerance to MTX who would be randomized in a 1:1 ratio to open‐label baricitinib or adalimumab (reference arm). Approximately an additional 20 patients who were MTX–inadequate responders (MTX‐IRs) or bDMARD‐IRs would be offered baricitinib.

The study design included a treatment period up to 24 weeks and an open‐label extension period up to 260 weeks. The data presented in this manuscript are for the treatment period up to 24 weeks only. A total of 30 patients were enrolled in the study (Supplemental Figure 1). One patient discontinued before receiving treatment. Of the 29 patients who received treatment, the first 10 patients that were MTX‐IRs and bDMARD naive were randomly assigned to baricitinib or adalimumab. The remaining 19 patients were MTX‐IRs and bDMARD‐IRs and were assigned to baricitinib treatment. Patients received oral baricitinib once daily at age‐based doses (9 to <18 years old: 4 mg; 2 to <9 years old: 2 mg). The reference arm of patients, who were MTX‐IRs and bDMARDs‐naive received adalimumab by subcutaneous injection every two weeks (20 or 40 mg based on weight). Trial registration number is NCT04088409.

### Outcomes

The primary efficacy endpoint was the proportion of responders at week 24, defined according to the SUN criteria as a two‐step decrease in the level of inflammation (AC cells) or decrease to zero through week 24 in the eye most severely affected at baseline. A Bayesian analysis was performed for the primary endpoint, based on prespecified success criteria (the posterior probability of the treatment response rate exceeding 57% is at least 80%). Response rates of a two‐step decrease in SUN criteria in baricitinib‐treated patients (who were MTX‐IRs and bDMARD‐IRs), baricitinib‐treated patients who were MTX‐IRs, and adalimumab‐treated patients who were MTX‐IRs are reported. Furthermore, two‐step and one‐step improvements in SUN criteria are reported over time. Secondary outcomes included change in SUN grade of cells in the AC, Pediatric American College of Rheumatology (PedACR) 30 criteria, PedACR50, PedACR70, PedACR90, and PedACR100 at week 24.^25^ Safety evaluations of baricitinib in pediatric patients with JIA‐U or ANA‐positive uveitis included treatment‐emergent adverse events (TEAEs). TEAEs were summarized using descriptive statistics.

### Statistical analysis

An open‐label Bayesian design was used for this study to maximize the benefit/risk balance for trial participants and to overcome the statistical inference challenge for small sample‐size studies. A Bayesian analysis determined if the study would be considered a positive study or a negative study. The study was considered positive if the posterior probability was at least 80% that the baricitinib treatment response would be >57%; otherwise, the study was considered negative.

A Bayesian posterior probability was calculated based on the number of observed responders who completed or had the opportunity to complete 24 weeks of the study. The details of the calculation of the posterior probability are as follows:

Let *X*_i_ denote the response of the 𝑖th patient, where *X*_i_|θ ∼ *Bern*(θ) and θ ∼ *Bet*(1,1) Then,

$$Y^{\text{stage}3}=\sum_{I=1}^{30}X_{i}$$

The Bayesian posterior probability for study success at the end of study (Stage 3) was calculated as

pr(θ>0.57│γstage3) = Pr(γstage3│θ>0.57).Pr(θ>0.57)Pr(γstage3)

The primary endpoint was imputed using modified NonResponder Imputation (mNRI). Of note, usage of an mNRI method can be justified based on the composite strategy (ICH E9[R1]) for handling intercurrent events.

In a composite strategy with NRI, a patient is defined as a responder only if (1) they meet the clinical requirements for response at the predefined time, and (2) they remain on the assigned study treatment; failing either criterion by definition makes them a nonresponder. For Study JAHW, this definition was modified such that, if either one of (1) or (2) criterion failed, the patient data would be reviewed by the Clinical Endpoints Committee (CEC) for further responder evaluation. The primary efficacy outcome variable, that is, the proportion of responders at week 24, in which response is defined according to the SUN criteria as a two‐step decrease in the level of inflammation (AC cells) or decrease to zero in the eye most severely affected at baseline, was imputed using mNRI.

Patients were thus considered nonresponders for the mNRI analysis if they did not meet the clinical response criteria or entirely missed the visit at the analysis time due to lack of efficacy. If the primary efficacy outcome variable data were missing for reasons other than this, available preceding data from patients with missing data were reviewed by an independent and external CEC, as outlined in the CEC charter. The CEC ensured that patients with missing data were evaluated in a consistent manner. The CEC considered the complete patient history and reasons for missing data in order to determine whether responder or nonresponder status was appropriate on a case‐by‐case basis.

Continuous data are summarized in terms of the observed mean, SD, minimum, maximum, and median. For categorical variables, NRI was used with 95% confidence intervals.

### Ethics

This trial was conducted in accordance with the ethical principles of the Declaration of Helsinki and Good Clinical Practice guidelines. The design was approved by the European Medicines Agency's Paediatric Committee (PDCO; EudraCT 2019‐000119‐10) and by various country‐level regulatory agencies and ethical review boards, including the CEIm del Hospital Universitario y Politécnico la Fe (Valencia, Spain), Comitato Etico San Martino (Genoa, Italy), Comitato Etico Milan Area 2 (Milan, Italy), CPP Nord Ouest IV—Lille (Lille, France), Landesamt für Gesundheit und Soziales (Berlin, Germany), and North West–Liverpool Central Research Ethics Committee (Liverpool, United Kingdom). All parents or legal guardians and patients (as appropriate) provided written informed consent and assent, respectively. Patient confidentiality will be maintained in accordance with the data privacy statement in the informed consent form.

### Data availability statement

Lilly provides access to all individual participant data collected during the trial, after anonymization, with the exception of pharmacokinetic or genetic data. Data are available to request six months after the indication studied has been approved in the US and European Union and after primary publication acceptance, whichever is later. No expiration date of data requests is currently set once data are made available. Access is provided after a proposal has been approved by an independent review committee identified for this purpose and after receipt of a signed data sharing agreement. Data and documents, including the study protocol, statistical analysis plan, clinical study report, blank or annotated case report forms, will be provided in a secure data sharing environment. For details on submitting a request, see the instructions provided at www.vivli.org.

## RESULTS

Recruitment to the trial proved challenging, particularly the subpopulation of patients who were MTX‐IRs to be randomized to baricitinib or adalimumab (reference arm), because only 10 of the planned 20 patients were recruited. Of the 29 patients receiving treatment, 24 patients (82.8%) received baricitinib and 5 patients (17.2%) received adalimumab (Table 1 and Supplemental Table 1). Of the baricitinib‐treated patients, 19 (79.2%) were MTX‐IRs and bDMARD‐IRs, and 5 (20.8%) were MTX‐IRs alone (Table 2). At baseline, for patients receiving baricitinib (n = 24), the median age was 12 years, 58.3% were female, and the vast majority were White (Table 1).

**Table 1:** Baseline demographics of the baricitinib‐ and adalimumab‐treated cohorts*

Treatment	Baricitinib (N = 24)	Adalimumab (N = 5)
Age, median (min, max), y	12.0 (6, 17)	7.0 (4, 9)
Weight, median (min, max), kg	47.2 (17.3, 92.1)	22.3 (17.0, 40.9)
Height, median (min, max), cm	155.9 (107.0, 179.1)	123.5 (103.5, 138.1)
BMI, median (min, max)	19.3 (14.6, 33.5)	16.0 (14.6, 21.5)
Female, n^a^ (%)	14.0 (58.3)	5 (100.0)
Male, n^a^ (%)	10.0 (41.7)	0 (0)
Race, n^a^ (%)		
White	20.0 (100.0)	4.0 (80.0)
Black or African American	0 (0)	1.0 (20.0)
Missing	4.0 (0)	0 (0)
Ethnicity, n^a^ (%)		
Hispanic or Latino	2.0 (8.7)	0 (0)
Not Hispanic or Latino	12.0 (52.2)	4.0 (80.0)
Not reported	9.0 (39.1)	1.0 (20.0)
Missing	1.0 (0)	0 (0)

* BMI, body mass index; N, number of patients in population.

^a^ Number of patients with non‐missing data, used as denominator.

**Table 2:** Baseline disease characteristics of the baricitinib‐treated cohort*

Disease characteristic	Baricitinib (N = 24)		
	Total (N = 24)	MTX‐IRs and bDMARD‐IRs (N = 19)	MTX‐IRs (N = 5)
JIA‐U and ANA+, n (%)	8 (33.3)	6 (31.6)	2 (40.0)
JIA‐U and ANA−, n (%)	12 (50.0)	10 (52.6)	2 (40.0)
ANA+ without JIA, n (%)	4 (16.7)	3 (15.8)	1 (20.0)
Time since uveitis diagnosis (y), median (min, max)	3.9 (0, 15)	5.7 (0, 15)	2.8 (0, 5)
Number of active joints, mean (SD)	0.5 (1.34)	0.7 (1.5)	0
Number of joints with limited range of motion, mean (SD)	0.3 (0.83)	0.3 (0.84)	0.4 (0.89)
AC SUN grading in the most severely affected eye, SUN grade, n (%)			
0.5+	1 (4.2)	0	1 (20)
1+	7 (29.2)	5 (26.3)	2 (40)
2+	7 (29.2)	5 (26.3)	2 (40)
3+	4 (16.7)	4 (21.1)	0
4+	5 (20.8)	5 (26.3)	0
Physician's global assessment,^a^ mean (SD)	3.9 (3.23)	4.2 (3.07)	3.0 (3.98)
Parent's global assessment,^b^ mean (SD)	31.8 (27.77)	34.0 (28.27)	24.0 (27.30)
CHAQ Physical Function (score), mean (SD)	0.3 (0.50)	0.4 (0.53)	0.2 (0.34)
ESR, mean (SD)	18.6 (27.85)	21.5 (30.14)	6.0 (8.04)
JADAS‐27, mean (SD)	8.3 (4.38)	8.7 (4.59)	6.5 (3.21)
Prior MTX use, n (%)	24 (100.0)	19 (100.0)	5 (100.0)
Prior bDMARD use, n (%)	19 (79.2)	19 (100.0)	0
Number of prior bDMARD used, n (%)			
1	13 (54.2)	13 (68.4)	0
2	3 (12.5)	3 (15.8)	0
>2	3 (12.5)	3 (15.8)	0

* AC, anterior chamber; ANA, anterior antinuclear antibody; bDMARD, biologic disease‐modifying antirheumatic drug; CHAQ, Childhood Health Assessment Questionnaire; ESR, erythrocyte sedimentation rate; IR, inadequate responder; JADAS, Juvenile Arthritis Disease Activity Score; JIA‐U, juvenile idiopathic arthritis–associated uveitis; MTX, methotrexate; N, number of patients in population; n, number of patients in the specified category; SD, standard deviation; SUN, Standardization of Uveitis Nomenclature.

^a^ Physician's Global Assessment of Disease Activity.

^b^ Parent's Global Assessment of Well‐Being.

Of the 19 patients that were bDMARD‐IRs, 13 patients (68.4%) had prior experience with one bDMARD, 3 patients (15.8%) had prior experience with two bDMARDs, and 3 patients (15.8%) had prior experience with more than two bDMARDs (Table 2). Regarding disease characteristics, 20 patients had JIA‐U and 4 had ANA‐positive chronic anterior uveitis without JAI‐U (Table 2). Of the 24 patients, 15 treated with baricitinib completed 24 weeks of the study.

For the primary outcome of a two‐step decrease in SUN criteria in the baricitinib group, 33% of patients achieved a response at week 24, resulting in 1.03% posterior probability of a response rate of >57% (Table 3); therefore, the study success criteria were not met. For baricitinib‐treated patients who were MTX‐IRs and bDMARD‐IRs, 36.8% of patients were responders. Additionally of the patients that were MTX‐IRs, 80% of adalimumab‐treated patients responded and 20% of baricitinib‐treated patients were responders.

**Table 3:** 2‐Step improvement in SUN grade of cells in the AC response rate at week 24 for the baricitinib‐treated and adalimumab‐treated cohorts*

	Baricitinib			Adalimumab (MTX‐IRs) N = 5
	Total N = 24	MTX‐IRs and bDMARD‐IRs N = 19	MTX‐IRs N = 5	
Response at wk 24, n (%)	8 (33.0)	7 (36.8)	1 (20.0)	4 (80.0)
Posterior probability of response rate >57%	1.03%	N/A	N/A	N/A

* AC, anterior chamber; bDMARD, biologic disease‐modifying antirheumatic drug; IR, inadequate responder; MTX, methotrexate; N, number of patients in population; n, number of patients in the specified category; N/A, not applicable; SUN, Standardization of Uveitis Nomenclature.

With regard to a step improvement in the SUN criteria, for baricitinib‐treated patients, there was a greater proportion of patients who achieved a one‐step improvement compared to a two‐step improvement across all time points (Table 4). Baricitinib displayed a rapid onset of effect with 70.8% of patients achieving a one‐step improvement at week 4. Although this high effect was not sustained, 45.8% of patients still achieved a one‐step improvement at week 24.

**Table 4:** Response rate of 1‐step and 2‐step improvement in SUN grade of cells in the AC for the baricitinib‐treated cohort*

Week	Baricitinib (N = 24)	
	SUN criteria 1‐Step improvement, n (%)	SUN criteria 2‐Step improvement, n (%)
4	17 (70.8)	9 (37.5)
8	14 (58.3)	7 (29.2)
12	16 (66.7)	6 (25.0)
16	15 (62.5)	10 (41.7)
20	14 (58.3)	11 (45.8)
24	11 (45.8)	8 (33.3)

* AC, anterior chamber; N, number of patients in population; n, number of patients in the specified category; SUN, Standardization of Uveitis Nomenclature.

For the secondary outcomes, at week 24, baricitinib‐treated patients had a mean change in SUN grade of cells in the AC of −1.47 (SD: 1.55) in the most severely affected eye and −0.27 (SD: 0.80) in the less severely affected eye (Supplemental Table 2). With regard to PedACR, at week 24, 29.2% of patients treated with baricitinib achieved PedACR30, and 20.8%, 20.8%, 8.3%, and 4.2% of patients achieved PedACR50, PedACR70, PedACR90, and PedACR100, respectively (Supplemental Table 3). Adalimumab‐treated patients’ secondary outcomes are reported in Supplemental Tables 4–7.

The overall safety data are shown in Table 5. The most frequent TEAE reported with baricitinib treatment was infections and infestations in 11 patients (45.8%). Two serious adverse events were reported during the trial (uveitis and intentional overdose of paracetamol), and one discontinuation from the study was due to an adverse event (worsening of uveitis). In the baricitinib group, 83.3% of patients reported at least one TEAE of which 41.7% were mild, 29.2% were moderate, and 12.5% were severe. Detailed lists of TEAEs are shown in Supplemental Tables 7–8.

**Table 5:** Treatment‐emergent adverse events occurring in at least 2 patients for the 24‐week safety population for the baricitinib‐treated cohort*

Treatment‐emergent adverse events	Baricitinib (N = 24), n (%)
Infections and infestations	11 (45.8)
COVID‐19	5 (20.8)
Nasopharyngitis	4 (16.7)
Upper respiratory tract infection	2 (8.3)
Gastrointestinal disorders	9 (37.5)
Nausea	5 (20.8)
Vomiting	4 (16.7)
Abdominal pain upper	3 (12.5)
Diarrhea	2 (8.3)
Respiratory, thoracic, and mediastinal disorders	7 (29.2)
Cough	4 (16.7)
Oropharyngeal pain	4 (16.7)
Eye disorders	6 (25.0)
Uveitis	2 (8.3)
General disorders and administration site conditions	5 (20.8)
Pyrexia	5 (20.8)
Musculoskeletal and connective tissue disorders	5 (20.8)
Arthralgia	2 (8.3)
Nervous system disorders	5 (20.8)
Headache	4 (16.7)
Injury, poisoning, and procedural complications	4 (16.7)
Ligament sprain	2 (8.3)
Investigations	4 (16.7)
Skin and subcutaneous tissue disorders	3 (12.5)
Acne	2 (8.3)

* COVID‐19, coronavirusdisease 2019; N, number of patients in population; n, number of patients in the specified category.

## DISCUSSION

Although there is a pressing need to provide a novel therapeutic approach for children with JIA‐U refractory to both MTX and bDMARDs, head‐to‐head or noninferiority studies are not possible for pediatric rheumatic diseases due to the rarity of diseases and the associated sample sizes that would be required. Historical comparisons with other placebo‐controlled trials do not address the key issue that access to care and therapeutic paradigms change every couple of years. This trial presents a novel approach through the inclusion of a reference arm of a standard of care biologic agent, which helps address issues important to patients and caregivers and clinicians.

In line with this, MTX‐IR patients and their parents showed a low interest in study enrollment due to the known efficacy of adalimumab and its known availability and recommendation as the first‐line therapy for MTX‐IR patients. Participation in the trial was also declined due to ethical concerns regarding random assignment to the experimental drug over the standard of care.

Additionally, daily dosing of baricitinib may also have been a limiting factor given that some parents prefer fortnightly injections over daily treatment. Enrollment completion in the MTX‐IR cohort was thus infeasible, and only 10 patients were randomized to the MTX‐IR cohort. The remaining 19 patients assigned to the baricitinib treatment were selected from the bDMARD‐IR cohort, as these patients were directly assigned to baricitinib without randomization. The Bayesian design use thus proved to be judicious to overcome statistical inference challenges linked to small sample sizes.

Although a limitation of the study is the small numbers due to recruitment challenge, especially for the randomized arm, strategized data analysis approaches based on the mNRI method and novel Bayesian design allowed us to overcome challenges related to clinical trials with small sample sizes. The mNRI method, in which missing data due to lack of efficacy are imputed as nonresponders, was used. The CEC reviewed available preceding data to determine responder or nonresponder status on a case‐by‐case basis. Any missing primary efficacy data for reasons other than lack of efficacy were thus imputed as nonresponders. However, there may be biases due to the nonnull risk of overestimating nonresponse rates, as patients who are lost to follow‐up are automatically considered nonresponders. This could lead to an underestimation of the treatment's efficacy. Lastly, imputation of missing data in such a small sample size has the potential to impact results, as each patient's data carry more weight in the overall analysis.

To decrease the impact of low patient recruitment in pediatric studies, a novel Bayesian design was used in order to overcome statistical inference challenges related to the small sample sizes. In the context of rare diseases—in which the number of eligible patients is inherently limited—a Bayesian approach enables more efficient use of data that can realistically be collected. Specifically, it allows for the incorporation of prior information (eg, expert opinion, historical data from related trials) to supplement limited trial data and to draw probabilistic conclusions about treatment effects even when sample sizes are small. This stands in contrast to traditional frequentist approaches, which often require larger sample sizes to achieve adequate power. Thus, although Bayesian analysis does not solve recruitment limitations, it provides a statistical framework that makes small‐sample trials feasible and interpretable when recruitment is constrained.^26^

Overall, in this study, most patients treated with baricitinib primarily had complex uveitis that failed to be controlled by one or several targeted biologic treatments including adalimumab (Supplemental Table 2). Of note, the adalimumab and baricitinib MTX‐IR cohorts had a slightly less severe distribution of AC cell count (Table 2 and Supplemental Table 9) but, overall, similar to the SYCAMORE study population (Table 1).^11^ On the other hand, the MTX‐IR and bDMARD‐IR cohort had a considerably more severe AC cell distribution, with a higher percentage of patients having 3+ and 4+ grades.

Additionally, in patients who are difficult to treat, the severity of disease and previous failure of response to therapy would make it harder to achieve disease control. In line with this, in a previous case study of patients with JIA‐U who were refractory to prior conventional DMARDs and bDMARDs and subsequently treated with JAK inhibitors, patients showed improvement of uveitis, which was defined as a reduction of intraocular inflammation according to SUN criteria. However, the mean follow‐up period for this case study was 7 months (range 4–13 months)^19^; therefore, a 24 week follow‐up for the primary outcome presented here may have underestimated the time for onset of efficacy in difficult‐to‐treat MTX‐IR and bDMARD‐IR patients.

Although the study success criteria were not met, it is noted that 36.8% of baricitinib‐treated patients that were MTX‐IRs and bDMARD‐IRs achieved the primary efficacy outcome. Moreover, of the patients that were MTX‐IRs, 80% of adalimumab‐treated patients responded and 20% of baricitinib‐treated patients were responders.

It is not possible to attribute the different outcomes to baseline characteristics in terms of AC cell distribution. A potential explanation to the greater response rate observed in patients who had failed both MTX and bDMARD than in patients who had failed MTX alone is that these patients may exhibit distinct disease mechanisms. In line with this, for bio‐naive patients with mild–moderate disease, TNF seems to be the primary driver of inflammation, which would explain the good response rate obtained with adalimumab. On the other hand, for TNF refractory patients with moderate–severe disease, baricitinib does seem to work, coherently with APTITUDE results.^17^ Another plausible factor to explain these results could be the influence of study design and patient selection criteria on the observed outcomes. The inclusion of biologic‐IR patients was driven by an unmet clinical need, whereas patients who had failed MTX monotherapy and were subsequently assigned to baricitinib in an open‐label setting may have had a preference for adalimumab. This does not exclude the possibility of bias within an already limited sample size.

Although the primary endpoint was not met, benefits were observed with baricitinib treatment, given that across all time points, a greater proportion of patients achieved a one‐step improvement. These results thus support previous findings obtained in small series of patients with JAK inhibitors being beneficial for the treatment of patients with JIA‐U.^18^, ^19^, ^20^, ^21^

In this JUVE‐BRIGHT study, the safety profile of baricitinib was found to be consistent with the established baricitinib indications in pediatric and adult patients.^22^, ^23^, ^24^ Baricitinib also offers the advantage of being an oral drug, which may increase compliance in juvenile patients. Given that most patients had complex uveitis with a lack of response to one or more targeted treatments including adalimumab and only a third of patients responded to baricitinib, it may offer clinicians with additional information on treatment approaches for children with JIA‐U who have failed both MTX and adalimumab. Although this study did not meet the primary outcome, the data provide additional information for the treatment of children with JIA‐U refractory to both MTX and bDMARDs. Overall, results from JUVE‐BRIGHT demonstrated that small portion of patients with JIA‐U or chronic anterior ANA‐positive uveitis that were MTX‐IRs and bDMARD‐IRs did achieve clinical response, which may be informative for clinicians to consider when treating for children who fail MTX and adalimumab.

## AUTHOR CONTRIBUTIONS

All authors contributed to at least one of the following manuscript preparation roles: conceptualization AND/OR methodology, software, investigation, formal analysis, data curation, visualization, and validation AND drafting or reviewing/editing the final draft. As corresponding author, Mr Ramanan confirms that all authors have provided the final approval of the version to be published, and takes responsibility for the affirmations regarding article submission (eg, not under consideration by another journal), the integrity of the data presented, and the statements regarding compliance with institutional review board/Declaration of Helsinki requirements.

## ROLE OF THE STUDY SPONSOR

Eli Lilly and Company had sole responsibility of study design, collection of data, analysis and interpretation of the data. Eli Lilly and Company did involve clinical experts as key consultants in the design of the study as well as interpretation of the data. Eli Lilly and Company was responsible for drafting the manuscript and submitting it for publication.

## Supporting information


**Disclosure Form**:

**Data S1** Supporting Information
