# Supplementary material for: Effectiveness and Safety of Baricitinib for Juvenile Idiopathic Arthritis–Associated Uveitis or Chronic Anterior Antinuclear Antibody–Positive Uveitis
Source: Arthritis Care Res (Hoboken). 2026 Feb 1;78(4):431–8. doi: 10.1002/acr.25644 (PMC13034097; doi:10.1002/acr.25644)
Supplement: Supplementary file 2 — Data S1 Supporting Information [file ACR-78-431-s002.docx]

**Supplementary Material**


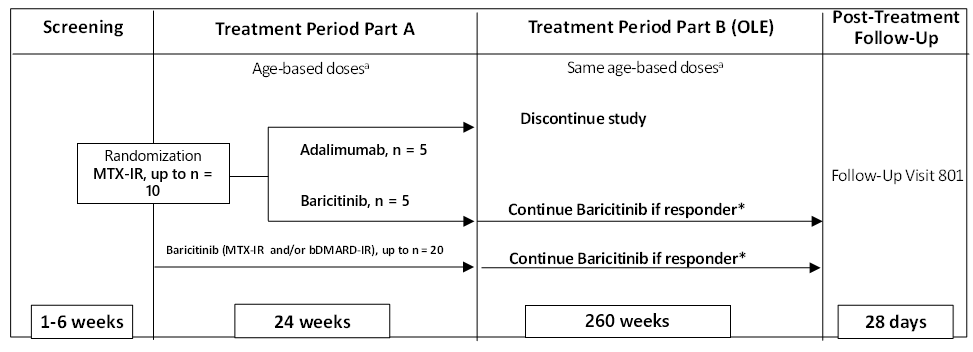


Supp Figure 1: Study design based on enrolled patients

Abbreviations: bDMARD-IR = biologic disease‑modifying antirheumatic drug-inadequate responder; MTX-IR = methotrexate inadequate responder; n = number of patients; OLE = open‑label extension.

^a^Patients ≥6 to <12 years old assigned to baricitinib have the option of receiving the oral suspension or tablets. Patients >12 years old assigned to baricitinib will receive tablets. Patients assigned to adalimumab weighing <30 kg will receive 20 mg, and those ≥30 kg will receive 40 mg.

Supp Table 1: Baseline demographics, adalimumab-treated cohort

| **Treatment** | **Adalimumab (N=5)** | |
| --- | --- | --- |
|  | **Median** | **Min, Max** |
| Age (years) | 7 | 4, 9 |
| Weight (kg) | 22.3 | 17.0, 40.9 |
| Height (cm) | 123.5 | 103.5, 138.1 |
| BMI | 16.0 | 14.6, 21.5 |
| Female, n (%) | 5 | 100 |
| Male, n (%) | 0 | 0 |
| **Race, n (%)** | | |
| White | 4 | 80.0 |
| Black or African American | 1 | 20.0 |
| Missing | 0 | 0.0 |
| **Ethnicity, n (%)** | | |
| Hispanic or Latino | 0 | 0 |
| Not Hispanic or Latino | 4 | 80.0 |
| Not Reported | 1 | 20.0 |
| Missing | 0 | 0 |

Supp Table 2: Change in SUN grade of cells in the anterior chamber through Week 24 in the most and less severely affected eye baricitinib-treated cohort

| **Mean (SD)** | **Baricitinib (N=24)** | |
| --- | --- | --- |
|  | **Most Severely Affected Eye** | **Less Severely Affected Eye** |
| Week 4 | n=24 | n=24 |
|  | -1.17 (1.090) | 0.21 (1.250) |
| Week 8 | n=22 | n=22 |
|  | -1.14 (1.283) | -0.36 (1.093) |
| Week 12 | n=21 | n=21 |
|  | -1.29 (1.384) | -0.14 (1.153) |
| Week 16 | n=18 | n=18 |
|  | -1.72 (1.447) | -0.28 (1.406) |
| Week 20 | n=16 | n=16 |
|  | -1.56 (1.094) | -0.56 (0.964) |
| Week 24 | n=15 | n=15 |
|  | -1.47 (1.552) | -0.27 (0.799) |

Abbreviations: N = number of patients in population; n = number of patients in the specified category; SD = standard deviation; SUN = Standardization of Uveitis Nomenclature.

Supp Table 3: PedACR response rates at Week 24 baricitinib-treated cohort

| **n (%)** | **Baricitinib (N=24)** |
| --- | --- |
| PedACR 30 Response | 7 (29.2) |
| PedACR 50 Response | 5 (20.8) |
| PedACR 70 Response | 5 (20.8) |
| PedACR 90 Response | 2 (8.3) |
| PedACR 100 Response | 1 (4.2) |

Abbreviations: N = number of patients in population; n = number of patients in the specified category; PedACR = Paediatric American College of Rheumatology.

Supp Table 4: Response Rate of 1-Step and 2-Step improvement in SUN Grade of Cells in the Anterior Chamber adalimumab-treated cohort

| **n (%)** | **Adalimumab (N=5)** | |
| --- | --- | --- |
|  | **SUN Criteria 1-Step Improvement** | **SUN Criteria 2-Step Improvement** |
| Week 4 | 3 (60.0) | 1 (20.0) |
| Week 8 | 2 (40.0) | 2 (40.0) |
| Week 12 | 3 (60.0) | 3 (60.0) |
| Week 16 | 3 (60.0) | 3 (60.0) |
| Week 20 | 3 (60.0) | 3 (60.0) |
| Week 24 | 4 (80.0) | 4 (80.0) |

Abbreviations: N = number of patients in population; n = number of patients in the specified category; SUN = Standardization of Uveitis Nomenclature.

Supp Table 5: Change in SUN grade of cells in the anterior chamber through Week 24 in the most and less severely affected eye adalimumab-treated cohort

| **Mean (SD)** | **Adalimumab (N=5)** | |
| --- | --- | --- |
|  | **Most severely affected eye** | **Less severely affected eye** |
| Week 4 | n=4 | n=4 |
|  | -1.25 (1.258) | -0.50 (0.577) |
| Week 8 | n=4 | n=4 |
|  | -1 (1.155) | 0.00 (1.414) |
| Week 12 | n=4 | n=4 |
|  | -1.75 (1.258) | -0.75 (0.500) |
| Week 16 | n=4 | n=4 |
|  | -1.00 (1.826) | -1.00 (0.816) |
| Week 20 | n=3 | n=3 |
|  | -2.33 (0.577) | -1.00 (0.000) |
| Week 24 | n=4 | n=4 |
|  | -2.25 (0.500) | -1.25 (0.500) |

Abbreviations: N = number of patients in population; n = number of patients in the specified category; SD = standard deviation.

Supp Table 6: PedACR response rates at Week 24 adalimumab-treated cohort

| **n (%)** | **Adalimumab (N=5)** |
| --- | --- |
| PedACR 30 Response | 0 (0.0) |
| PedACR 50 Response | 0 (0.0) |
| PedACR 70 Response | 0 (0.0) |
| PedACR 90 Response | 0 (0.0) |
| PedACR 100 Response | 0 (0.0) |

Abbreviations: N = number of patients in population; n = number of patients in the specified category; PedACR = Paediatric American College of Rheumatology.

*Supp Table 7: Treatment-Emergent Adverse Events adalimumab-treated cohort*

| **Treatment-Emergent Adverse Events, n (%)** | **Adalimumab (N=5)** |
| --- | --- |
| **Infections and infestations** | 3 (60.0) |
| COVID-19 | 1 (20.0) |
| Nasopharyngitis | 1 (20.0) |
| Upper respiratory tract infection | 0 (0) |
| Bronchitis | 0 (0) |
| Epstein-Barr virus infection | 0 (0) |
| Eye infection | 0 (0) |
| Fungal disease carrier | 0 (0) |
| Gastrointestinal infection | 0 (0) |
| Herpes simplex | 0 (0) |
| Influenza | 0 (0) |
| Lower respiratory tract infection | 0 (0) |
| Pharyngitis | 0 (0) |
| Rhinitis | 0 (0) |
| Tonsillitis | 0 (0) |
| Urinary tract infection | 1 (20.0) |
| Oral herpes | 1 (20.0) |
| Otitis media | 1 (20.0) |
| **Gastrointestinal disorders** | 1 (20.0) |
| Nausea | 0 (0) |
| Vomiting | 0 (0) |
| Abdominal pain upper | 1 (20.0) |
| Diarrhoea | 0 (0) |
| Abdominal pain | 0 (0) |
| Lip swelling | 0 (0) |
| Stomatitis | 0 (0) |
| **Respiratory, thoracic and mediastinal disorders** | 0 (0) |
| Cough | 0 (0) |
| Oropharyngeal pain | 0 (0) |
| Dyspnoea | 0 (0) |
| Oropharyngeal blistering | 0 (0) |
| **Eye disorders** | 1 (20.0) |
| Uveitis | 0 (0) |
| Chalazion | 0 (0) |
| Eye irritation | 0 (0) |
| Eye pain | 0 (0) |
| Glaucoma | 0 (0) |
| Retinal vasculitis | 0 (0) |
| Vitreous floaters | 0 (0) |
| Macular oedema | 1 (20) |
| **General disorders and administration site conditions** | 2 (40.0) |
| Pyrexia | 1 (20) |
| Adverse drug reaction | 1 (20) |
| Asthenia | 0 (0) |
| Fatigue | 0 (0) |
| Illness | 1 (20) |
| **Musculoskeletal and connective tissue disorders** | 2 (40.0) |
| Arthralgia | 0 (0) |
| Arthritis | 0 (0) |
| Back pain | 0 (0) |
| Pain in extremity | 0 (0) |
| Pain in jaw | 0 (0) |
| Bone development abnormal | 1 (20.0) |
| Juvenile idiopathic arthritis | 1 (20.0) |
| **Nervous system disorders** | 0 (0) |
| Headache | 0 (0) |
| Dizziness | 0 (0) |
| **Injury, poisoning and procedural complications** | 1 (20.0) |
| Ligament sprain | 0 (0) |
| Contusion | 0 (0) |
| Fall | 0 (0) |
| Intentional overdose | 0 (0) |
| Injection related reaction | 1 (20.0) |
| **Investigations** | 1 (20.0) |
| Basophil count decreased | 0 (0) |
| Blood cholesterol increased | 0 (0) |
| Blood creatine phosphokinase increased | 0 (0) |
| Blood iron increased | 0 (0) |
| Blood triglycerides increased | 1 (20.0) |
| Body temperature increased | 0 (0) |
| Cell marker decreased | 0 (0) |
| Intraocular pressure increased | 0 (0) |
| Iron binding capacity total increased | 0 (0) |
| Monocyte count increased | 0 (0) |
| Neutrophil count decreased | 1 (20.0) |
| SARS-CoV-2 test positive | 0 (0) |
| T-lymphocyte count decreased | 0 (0) |
| White blood cell count decreased | 0 (0) |
| Alanine aminotransferase increased | 1 (20.0) |
| Aspartate aminotransferase increased | 1 (20.0) |
| Bilirubin conjugated increased | 1 (20.0) |
| Blood bilirubin increased | 1 (20.0) |
| Eosinophil count increased | 1 (20.0) |
| Mean platelet volume decreased | 1 (20.0) |
| Platelet count increased | 1 (20.0) |
| **Skin and subcutaneous tissue disorders** | 0 (0) |
| Acne | 0 (0) |
| Rash maculo-papular | 0 (0) |
| **Blood and lymphatic system disorders** | 1 (20.0) |
| Neutropenia | 0 (0) |
| Iron deficiency anaemia | 1 (20.0) |
| **Ear and labyrinth disorders** | 0 (0) |
| Tinnitus | 0 (0) |
| **Metabolism and nutrition disorders** | 0 (0) |
| Decreased appetite | 0 (0) |
| **Neoplasms benign, malignant and unspecified (incl cysts and polyps)** | 0 (0) |
| Fibrous cortical defect | 0 (0) |
| **Psychiatric disorders** | 0 (0) |
| Anxiety | 0 (0) |
| Depression | 0 (0) |
| **Surgical and medical procedures** | 0 (0) |
| Glaucoma drainage device placement | 0 (0) |

Abbreviations: COVID-19 = coronavirus disease 2019; N = number of patients in population; n = number of patients in the specified category.

*Supp Table 8: Treatment-Emergent Adverse Events baricitinib-treated cohort*

| **Treatment-Emergent Adverse Events, n (%)** | **Baricitinib (N=24)** |
| --- | --- |
| **Infections and infestations** | 11 (45.8) |
| COVID-19 | 5 (20.8) |
| Nasopharyngitis | 4 (16.7) |
| Upper respiratory tract infection | 2 (8.3) |
| Bronchitis | 1 (4.2) |
| Epstein-Barr virus infection | 1 (4.2) |
| Eye infection | 1 (4.2) |
| Fungal disease carrier | 1 (4.2) |
| Gastrointestinal infection | 1 (4.2) |
| Herpes simplex | 1 (4.2) |
| Influenza | 1 (4.2) |
| Lower respiratory tract infection | 1 (4.2) |
| Pharyngitis | 1 (4.2) |
| Rhinitis | 1 (4.2) |
| Tonsillitis | 1 (4.2) |
| Urinary tract infection | 1 (4.2) |
| Oral herpes | 0 (0) |
| Otitis media | 0 (0) |
| **Gastrointestinal disorders** | 9 (37.5) |
| Nausea | 5 (20.8) |
| Vomiting | 4 (16.7) |
| Abdominal pain upper | 3 (12.5) |
| Diarrhoea | 2 (8.3) |
| Abdominal pain | 1 (4.2) |
| Lip swelling | 1 (4.2) |
| Stomatitis | 1 (4.2) |
| **Respiratory, thoracic and mediastinal disorders** | 7 (29.2) |
| Cough | 4 (16.7) |
| Oropharyngeal pain | 4 (16.7) |
| Dyspnoea | 1 (4.2) |
| Oropharyngeal blistering | 1 (4.2) |
| **Eye disorders** | 6 (25) |
| Uveitis | 2 (8.3) |
| Chalazion | 1 (4.2) |
| Eye irritation | 1 (4.2) |
| Eye pain | 1 (4.2) |
| Glaucoma | 1 (4.2) |
| Retinal vasculitis | 1 (4.2) |
| Vitreous floaters | 1 (4.2) |
| Macular oedema | 0 (0) |
| **General disorders and administration site conditions** | 5 (20.8) |
| Pyrexia | 5 (20.8) |
| Adverse drug reaction | 1 (4.2) |
| Asthenia | 1 (4.2) |
| Fatigue | 1 (4.2) |
| Illness | 0 (0) |
| **Musculoskeletal and connective tissue disorders** | 5 (20.8) |
| Arthralgia | 2 (8.3) |
| Arthritis | 1 (4.2) |
| Back pain | 1 (4.2) |
| Pain in extremity | 1 (4.2) |
| Pain in jaw | 1 (4.2) |
| Bone development abnormal | 0 (0) |
| Juvenile idiopathic arthritis | 0 (0) |
| **Nervous system disorders** | 5 (20.8) |
| Headache | 4 (16.7) |
| Dizziness | 1 (4.2) |
| **Injury, poisoning and procedural complications** | 4 (16.7) |
| Ligament sprain | 2 (8.3) |
| Contusion | 1 (4.2) |
| Fall | 1 (4.2) |
| Intentional overdose | 1 (4.2) |
| Injection related reaction | 0 (0) |
| **Investigations** | 4 (16.7) |
| Basophil count decreased | 1 (4.2) |
| Blood cholesterol increased | 1 (4.2) |
| Blood creatine phosphokinase increased | 1 (4.2) |
| Blood iron increased | 1 (4.2) |
| Blood triglycerides increased | 1 (4.2) |
| Body temperature increased | 1 (4.2) |
| Cell marker decreased | 1 (4.2) |
| Intraocular pressure increased | 1 (4.2) |
| Iron binding capacity total increased | 1 (4.2) |
| Monocyte count increased | 1 (4.2) |
| Neutrophil count decreased | 1 (4.2) |
| SARS-CoV-2 test positive | 1 (4.2) |
| T-lymphocyte count decreased | 1 (4.2) |
| White blood cell count decreased | 1 (4.2) |
| Alanine aminotransferase increased | 0 (0) |
| Aspartate aminotransferase increased | 0 (0) |
| Bilirubin conjugated increased | 0 (0) |
| Blood bilirubin increased | 0 (0) |
| Eosinophil count increased | 0 (0) |
| Mean platelet volume decreased | 0 (0) |
| Platelet count increased | 0 (0) |
| **Skin and subcutaneous tissue disorders** | 3 (12.5) |
| Acne | 2 (8.3) |
| Rash maculo-papular | 1 (4.2) |
| **Blood and lymphatic system disorders** | 1 (4.2) |
| Neutropenia | 1 (4.2) |
| Iron deficiency anaemia | 0 (0) |
| **Ear and labyrinth disorders** | 1 (4.2) |
| Tinnitus | 1 (4.2) |
| **Metabolism and nutrition disorders** | 1 (4.2) |
| Decreased appetite | 1 (4.2) |
| **Neoplasms benign, malignant and unspecified (incl cysts and polyps)** | 1 (4.2) |
| Fibrous cortical defect | 1 (4.2) |
| **Psychiatric disorders** | 1 (4.2) |
| Anxiety | 1 (4.2) |
| Depression | 1 (4.2) |
| **Surgical and medical procedures** | 1 (4.2) |
| Glaucoma drainage device placement | 1 (4.2) |

Abbreviations: COVID-19 = coronavirus disease 2019; N = number of patients in population; n = number of patients in the specified category.

Supp Table 9: Baseline disease characteristics, adalimumab-treated cohort

| **Disease Characteristic** | **Adalimumab  (N=5)** |
| --- | --- |
| JIA-U and ANA+, n (%) | 2 (40.0) |
| JIA-U and ANA-, n (%) | 2 (40.0) |
| ANA+ without JIA, n (%) | 1 (20.0) |
| Time since uveitis diagnosis (years), median (min, max) | 2.3 (0, 6) |
| Number of active joints, mean (SD) | 0 |
| Number of joints with limited range of motion, mean (SD) | 0.8 (1.79) |
| Anterior chamber SUN grading in the most severely affected eye, SUN grade, n (%) |  |
| 0.5+ | 1 (20.0) |
| 1+ | 3 (60.0) |
| 2+ | 1 (20.0) |
| 3+ | 0 |
| 4+ | 0 |
| Physician’s global assessment^a^, mean (SD) | 1.5 (1.50) |
| Parent’s global assessment^b^, mean (SD) | 10.2 (16.24) |
| CHAQ Physical Function (score), mean (SD) | 0.6 (0.68) |
| ESR, mean (SD) | 8.5 (2.12) |
| JADAS-27, mean (SD) | 2.5 (0.71) |
| Prior MTX use, n (%) | 4 (80.0)^c^ |
| Prior bDMARD use, n (%) | 0 |
| Number of prior bDMARD used, n (%) |  |
| 1 | 0 |
| 2 | 0 |
| >2 | 0 |

Abbreviations: ANA+ = anterior antinuclear antibody positive; ANA- = anterior antinuclear antibody negative; bDMARD = biological

disease-modifying antirheumatic drugs; bDMARD-IR = bDMARD inadequate responder CHAQ = childhood Health Assessment

Questionnaire; ESR = erythrocyte sedimentation rate; JADAS = Juvenile Arthritis Disease Activity Score; JIA-U = juvenile idiopathic arthritis

associated uveitis; MTX = methotrexate; MTX-IR = MTX-inadequate responder; N = number of patients in population; n = number of

patients in the specified category; SD = standard deviation: SUN = Standardization of Uveitis Nomenclature.

aPhysician’s global assessment of Disease Activity.

bParent’s Global Assessment of Well-Being.

cSite did not update electronic case report form until after database lock occurred, but prior MTX usage was confirmed for patient. Hence, the patient is missing from the parameter = “yes”.
